# T cell subtypes and reciprocal inflammatory mediator expression differentiate *P*. *falciparum* memory recall responses in asymptomatic and symptomatic malaria patients in southeastern Haiti

**DOI:** 10.1371/journal.pone.0174718

**Published:** 2017-04-03

**Authors:** Jason S. Lehmann, Joseph J. Campo, Micheline Cicéron, Christian P. Raccurt, Jacques Boncy, Valery E. M. Beau De Rochars, Anthony P. Cannella

**Affiliations:** 1 Division of Infectious Diseases and Global Medicine, Department of Medicine, College of Medicine, University of Florida, Gainesville, Florida, United States of America; 2 Emerging Pathogen Institute, University of Florida, Gainesville, Florida, United States of America; 3 Antigen Discovery Inc., Irvine, California, United States of America; 4 Laboratoire National de Santé Publique (LNSP), Ministère de la Santé Publique et de la Population (MSPP), Port-au-Prince, Haiti; 5 Department of Health Services Research, Management and Policy, College of Public Health and Health Professions, University of Florida, Gainesville, Florida, United States of America; 6 Department of Molecular Genetics & Microbiology, College of Medicine, University of Florida, Gainesville, Florida, United States of America; Universidade Federal de Minas Gerais, BRAZIL

## Abstract

Asymptomatic *Plasmodium falciparum* infection is responsible for maintaining malarial disease within human populations in low transmission countries such as Haiti. Investigating differential host immune responses to the parasite as a potential underlying mechanism could help provide insight into this highly complex phenomenon and possibly identify asymptomatic individuals. We performed a cross-sectional analysis of individuals who were diagnosed with malaria in Sud-Est, Haiti by comparing the cellular and humoral responses of both symptomatic and asymptomatic subjects. Plasma samples were analyzed with a *P*. *falciparum* protein microarray, which demonstrated serologic reactivity to 3,877 *P*. *falciparum* proteins of known serologic reactivity; however, no antigen-antibody reactions delineating asymptomatics from symptomatics were identified. In contrast, differences in cellular responses were observed. Flow cytometric analysis of patient peripheral blood mononuclear cells co-cultured with *P*. *falciparum* infected erythrocytes demonstrated a statistically significant increase in the proportion of T regulatory cells (CD4^+^ CD25^+^ CD127^-^), and increases in unique populations of both NKT-like cells (CD3^+^ CD8^+^ CD56^+^) and CD8^mid^ T cells in asymptomatics compared to symptomatics. Also, CD38^+^/HLA-DR^+^ expression on γδ T cells, CD8^mid^ (CD56^-^) T cells, and CD8^mid^ CD56^+^ NKT-like cells decreased upon exposure to infected erythrocytes in both groups. Cytometric bead analysis of the co-culture supernatants demonstrated an upregulation of monocyte-activating chemokines/cytokines in asymptomatics, while immunomodulatory soluble factors were elevated in symptomatics. Principal component analysis of these expression values revealed a distinct clustering of individual responses within their respective phenotypic groups. This is the first comprehensive investigation of immune responses to *P*. *falciparum* in Haiti, and describes unique cell-mediated immune repertoires that delineate individuals into asymptomatic and symptomatic phenotypes. Future investigations using large scale biological data sets analyzing multiple components of adaptive immunity, could collectively define which cellular responses and molecular correlates of disease outcome are malaria region specific, and which are truly generalizable features of asymptomatic *Plasmodium* immunity, a research goal of critical priority.

## Introduction

Human malaria, caused by five parasite species of the genus, *Plasmodium*, accounted for 214 million cases worldwide with an estimated 500,000 deaths in 2015 [1]. Even though most of the morbidity and mortality are attributed to infection with *P*. *falciparum* in sub-Saharan Africa, there are many areas of the globe where malaria remains a major public health concern. Extreme poverty and a limited infrastructure had already affected the efficacy of control measures to a number of infectious diseases in the Republic of Haiti prior to 2010. Following the January 2010 earthquake, these issues worsened exponentially, and thus the public health response to numerous infectious diseases including malaria was further compromised.

Despite this prevailing obstacle, both Haiti and its eastern neighbor, the Dominican Republic, have mandated a strategy to eliminate malaria on the island of Hispaniola by the year 2020 [2]. There is a real potential to accomplish this due to the epidemiology of malaria in Hispaniola, as well as due to several advantages that would support the potential of malaria elimination on the island: 1) Haiti and the Dominican Republic have a lower rate of importation of malaria infections from other areas, compared to other malaria endemic countries [3–5]; 2) *P*. *falciparum* (Pf) is the one and only cause of endemic malaria on Hispaniola (there is a lack other *Plasmodium* species associated with human infection on the island) and thus elimination efforts are being concentrated on this one organism [4]; 3) chloroquine resistance to *P*. *falciparum* is rarely reported in Haiti, despite wide use as the standard of treatment for both Haiti and the Dominican Republic [5–7].

One of the true challenges to the elimination of malaria in both Hispaniola and around the globe is the complete prevention of parasite transmission, which absolutely requires the identification and treatment of asymptomatic malaria parasite carriers [8, 9]. This challenge is magnified in areas where there is low transmission of *Plasmodium* such as on Hispaniola. Currently, there is no international standard for the definition of asymptomatic malaria; it usually refers to the presence of both asexual blood stages and gametocytes of *Plasmodium* species without the presence of any acute manifestations of malaria (i.e. fever, abdominal pain and headache) [1, 9]. Due to the lack of clinical symptoms of malaria, there is no definitive way to identify these asymptomatic individuals, unless they are randomly identified via either microscopy or commercial rapid diagnostic tests (RDTs). However, submicroscopic infections in asymptomatic individuals cannot definitively be identified due to the limit of detection of these commercial RDTs. Immunological studies (cellular immune responses, chemokine/cytokine differences and various humoral responses) performed in other malaria endemic regions have been documented [10–16]. For asymptomatic infections [17, 18], the focus of most of these investigations has been on a single chemokine/cytokine or a particular cellular population percentage noted when comparing individuals with either asymptomatic vs. symptomatic malaria. Furthermore, only a few comprehensive investigations into the adaptive immunologic recall response to *Plasmodium* infection have been performed and nowhere has the humoral, cellular and chemokine/cytokine responses all been observed in one encompassing study.

**Fig 1:**
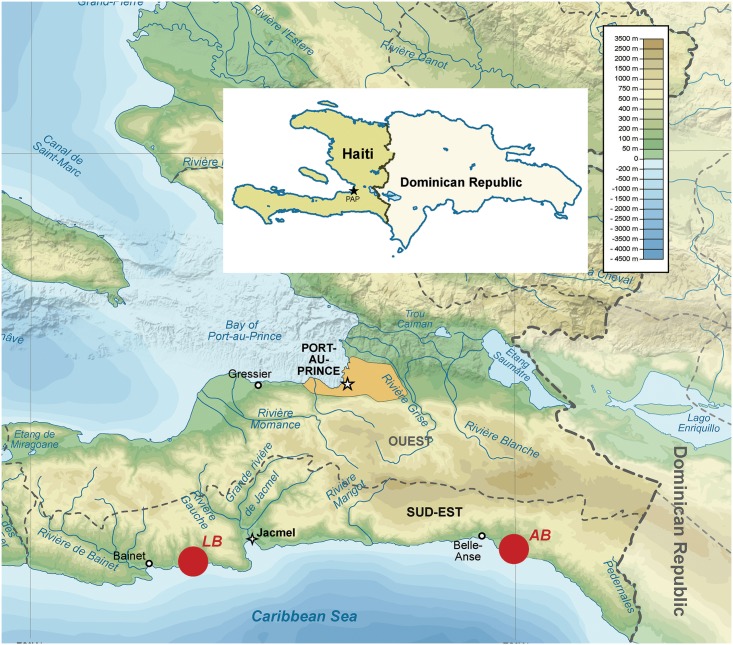
Map of the department of Sud-Est Haiti displaying sites of asymptomatic and symptomatic malaria patients were collected. Map of the Department of Sud-Est, Haiti with clinical sites La Brésilienne (LB) and Anse à Boeuf (AB) where individuals’ samples were collected from these two respective villages. The two sites were over 70 km apart in distance, and had poorly traversable roads between the two villages. Map of the island of Hispaniola (insert) displaying the two countries Haiti and the Dominican Republic is included. A topographic scale is provided for reference purposes. Maps were provided by Wikipedia and Wikimedia Commons, and were modified to display sample sites accurately.

The Republic of Haiti, for reasons noted above, provides a unique opportunity to explore the underlying immune profile differences between asymptomatic and symptomatic individuals infected with malaria, as well as possibly elucidating diagnostic clues to identifying these previously unknown asymptomatic cases. Therefore, the goal of this study was to perform a cross-sectional investigation into the adaptive immunological recall response to erythrocytic stages of an autologous *P*. *falciparum* strain in both asymptomatic and symptomatic cohorts from the Sud-Est department of Haiti (Fig 1). This cohort was previously identified by either microscopy or rapid diagnostic test (RDT), and all individuals were treated as to Haiti Ministry of Health mandates. The results of this study demonstrated some intriguing findings, such as decreased immune cell activation markers, the identification of two unique T cell populations, and reciprocal chemokine/cytokine expression patterns that distinctively characterize asymptomatic malaria infections, and distinguish them from the immune responses in patients with symptomatic malaria infections.

## Results

### Patient cohort

There were a total of 29 participants (Table 1), with both men and women enrolled in the study from an age range of 18 to 64 years, with a mean age of 32.5 years. Participants were previously classified as having symptomatic or asymptomatic malaria infections, based on clinician diagnosis of parasitemia (via either microscopy or RDT) and presence or absence of fever [19]. Samples were approximately evenly split between two villages in Haiti (Anse à Boeuf: n = 14; La Brésilienne: n = 15).

**Table 1:** Demographics of the participants from Sud-Est, Haiti.

		La Brésilienne	Anse a Boeuf
Symptomatics	15	7	8
Asymptomatics	14	8	6
Totals	29	15	14

### Protein microarray analysis of IgG and IgM serological reactivity to *P*. *falciparum (Pf)* antigens in symptomatic and asymptomatic malaria patients from the Sud-Est department, Haiti

**Fig 2:**
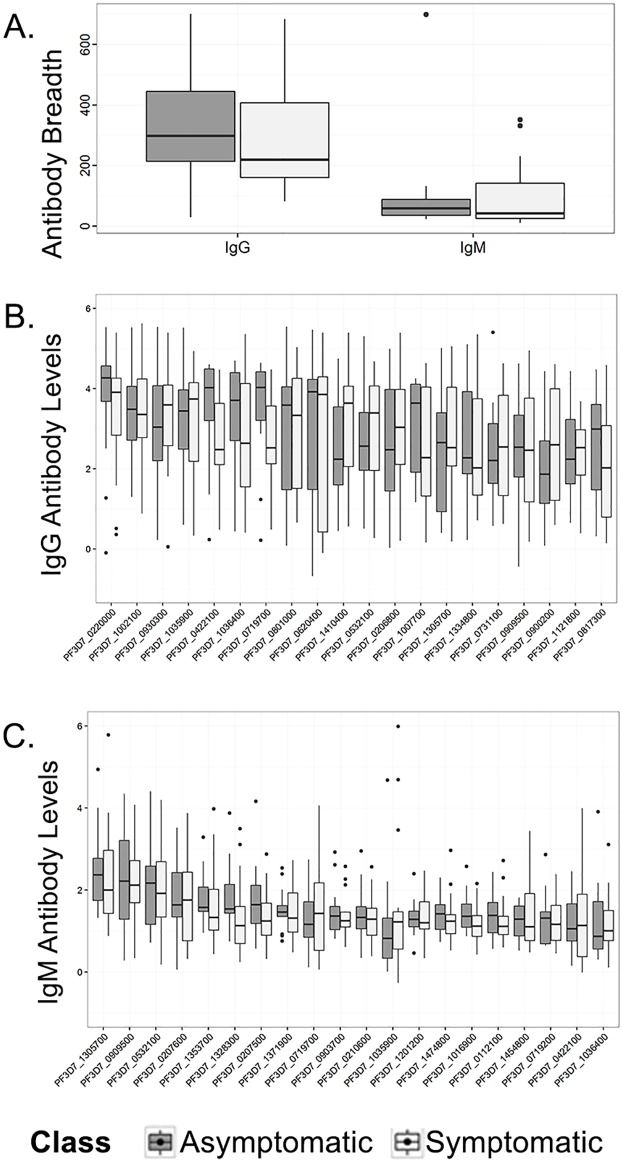
Results from Pf protein microarray analysis of IgG & IgM antibody repertoires from asymptomatic and symptomatic malaria patients in Sud-Est, Haiti. Plasma from asymptomatic (n = 16) and symptomatic (n = 13) malaria patients from the Sud-Est Department in Haiti were reacted with a partial Pf proteome microarrays. Arrays contained 3,877 peptide fragments from 2,704 unique genes, representing approximately 50% coverage of the Pf proteome. **(A)** Box plots representing antibody breadth score per patient for both IgG and IgM, described as sum of seropositive responses per individual. Breadth did not show an effect on probability of having symptoms. **(B)** Box plots of IgG responses of asymptomatic and symptomatic patients to top 20 antigens by magnitude. **(C)** Box plots of IgM responses of asymptomatic and symptomatic patients to top 20 antigens by magnitude. The magnitude of neither IgG nor IgM responses showed an effect on probability of having symptoms. **(A-C)** box lines represent the median and interquartile ranges, where the whiskers represent all points within 1.5 times the interquartile range, and points represent outliers.

Due to the established importance of humoral immunity during Pf infection, a comparative analysis of IgG and IgM Pf antibody targets from both Haitian cohorts (asymptomatic and symptomatic) was performed by reacting plasma samples from with a partial Pf proteome microarray [16, 20, 21]. Two outcomes were then tested in each group comparison: 1) antibody levels (“antibody magnitude”) for each antigen 2) the effect of seropositive responses for each individual (“antibody breadth”) on symptoms (Fig 2).

Analysis of IgG antibody breadth for all patients revealed that there were 960 reactive antigens out of 3877 total antigens on the chip. As a group, asymptomatic patients (n = 14) recognized a median total of 298 antigen targets (95% CI [214–446]), while symptomatic patients (n = 15) recognized a median total of 219, 95% CI [160–407], (Fig 2A). After correction for the false discovery rate, the difference between the two patient cohorts for antibody levels to individual antigens was non-significant (adjusted P-value all antigens > 0.05). IgM antibody breadth for all patients was comprised of 337 reactive antigens out of 3877 total antigens on the chip. The asymptomatic median seropositivity was 58 antigens, 95% CI [36–88], while the symptomatic median was 42 antigens, 95% CI [26–142], (Fig 2A). Statistical analyses of the differences between results were also found to be not significant after correction for the false discovery rate. Box plots representing group-wise comparisons between the global top 20 antibody responses for both isotypes in all patients based on the magnitude of the mean antibody levels are displayed graphically (Fig 2B and 2C), and listed in Tables 2 & 3. Similarly to the antibody breadth analysis, statistical testing of the magnitude (intensity of antibody response) was also not significant between the two groups (adjusted P-value all antigens > 0.05). (Fig 2A) Box plots representing antibody breadth score per patient for both IgG and IgM, described as sum of seropositive responses per individual. Breadth did not show an effect on probability of having symptoms (IgG P-value: 0.252; IgM P-value: 0.446). (Fig 2B) Box plots of IgG responses of asymptomatic and symptomatic patients to top 20 antigens by magnitude, and are listed in Table 2. (Fig 2C) Box plots of IgM responses of asymptomatic and symptomatic patients to top 20 antigens by magnitude, and are listed in Table 3. The magnitude of neither IgG nor IgM responses showed an effect on probability of having symptoms (adjusted P-value all antigens > 0.05). (Fig 2A–2C) box lines represent the median and interquartile ranges, the whiskers represent all points within 1.5 times the interquartile range, and points represent outliers.

**Table 2:** IgG: Top 20 *P*. *falciparum* antigens by magnitude recognized by patient sera.

Gene.ID	Description
PF3D7_0220000	Liver stage antigen 3 (LSA3)
PF3D7_1002100	PF70 protein (PF70)
PF3D7_0930300	Merozoite surface protein 1 (MSP1)
PF3D7_1035900	Probable protein, unknown function
PF3D7_0422100	Transmembrane emp24 domain-containing protein, putative
PF3D7_1036400	Liver stage antigen 1 (LSA1)
PF3D7_0719700	40S ribosomal protein S10, putative
PF3D7_0801000	Plasmodium exported protein (PHISTc), unknown function
PF3D7_0620400	Merozoite surface protein 10 (MSP10)
PF3D7_1410400	Rhoptry-associated protein 1 (RAP1)
PF3D7_0532100	Early transcribed membrane protein 5 (ETRAMP5)
PF3D7_0206800	Merozoite surface protein 2 (MSP2)
PF3D7_1007700	Transcription factor with AP2 domain(s) (ApiAP2)
PF3D7_1305700	Conserved Plasmodium protein, unknown function
PF3D7_1334800	MSP7-like protein (MSRP2)
PF3D7_0731100	Plasmodium exported protein (PHISTc), unknown function (GEXP11)
PF3D7_0909500	Subpellicular microtubule protein 1, putative (SPM1)
PF3D7_0900200	Rifin (RIF)
PF3D7_1121800	Peptidase, M16 family
PF3D7_0817300	Asparagine-rich antigen

**Table 3:** IgM: Top 20 *P*. *falciparum* antigens by magnitude recognized by patient sera.

Gene.ID	Description
PF3D7_1305700	Conserved Plasmodium protein, unknown function
PF3D7_0909500	Subpellicular microtubule protein 1, putative (SPM1)
PF3D7_0532100	Early transcribed membrane protein 5 (ETRAMP5)
PF3D7_0207600	Serine repeat antigen 5 (SERA5)
PF3D7_1353700	Conserved Plasmodium protein, unknown function
PF3D7_1328300	Conserved Plasmodium protein, unknown function
PF3D7_0207500	Serine repeat antigen 6 (SERA6)
PF3D7_1371900	Plasmodium exported protein, unknown function
PF3D7_0719700	40S ribosomal protein S10, putative
PF3D7_0903700	Alpha tubulin 1
PF3D7_0210600	Conserved Plasmodium protein, unknown function
PF3D7_1035900	Probable protein, unknown function
PF3D7_1201200	Plasmodium exported protein, unknown function
PF3D7_1474800	Proteosome subunit alpha type 1, putative
PF3D7_1016900	Early transcribed membrane protein 10.3 (ETRAMP10.3)
PF3D7_0112100	Conserved Plasmodium protein, unknown function
PF3D7_1454800	Conserved Plasmodium protein, unknown function
PF3D7_0719200	NIMA related kinase 4 (NEK4)
PF3D7_0422100	Transmembrane emp24 domain-containing protein, putative
PF3D7_1036400	Liver stage antigen 1 (LSA1)

Antibodies from both cohorts recognized protein antigens previously noted to generate humoral responses *in vivo*. These include Pf surface proteins such as merozoite surface proteins, MSPs, [22], Early transcribed membrane proteins, ETRAMPs, [23], and even Liver Stage Antigens, LSAs, [24]. There were also several putative proteins of unknown function that generated responses. In addition, there was no overlap in the top twenty antigens with regards to IgG and IgM reactivity with the exception of LSA-1, which was bound by both IgG and IgM antibodies. The results corroborate previous investigations in low endemic settings, in that there are numerous Pf antigens that elicit humoral responses [25, 26]; however, there was no demonstrable evidence that any of these Pf antigens were diagnostic in separating asymptomatic from symptomatic malaria patients in Haiti.

### Phenotyping T cell population dynamics during PBMC: *P*. *falciparum* schizont lysate co-culture using multiparametric flow cytometry

Since T cells are involved with the initiation of a memory response to protein antigens, we investigated the T cell arm of the adaptive immune response to the Pf asexual blood stages. Previous studies investigating patient groups from other geographical areas or in murine models have reported specific roles for regulatory CD4 T cells [27, 28], CD8 T cells [29, 30], CD4 T cells [31, 32], and γδ T cells [10, 33, 34] in the immune response to malaria infection, as well as generalized lymphocyte perturbations in patients from endemic areas of transmission [35–37]. To assess memory T cell responses, 5 x 10^5^ PBMCs from symptomatic (n = 6), asymptomatic (n = 9), were co-cultured for six days with uninfected erythrocytes or schizont lysate from the local Haitian *P*. *falciparum* strain H1064 at a schizont to PBMC ratio of 2:1. After stimulation, PBMCs were subjected to multi-parametric flow cytometric analysis (S1 Fig).

**Fig 3:**
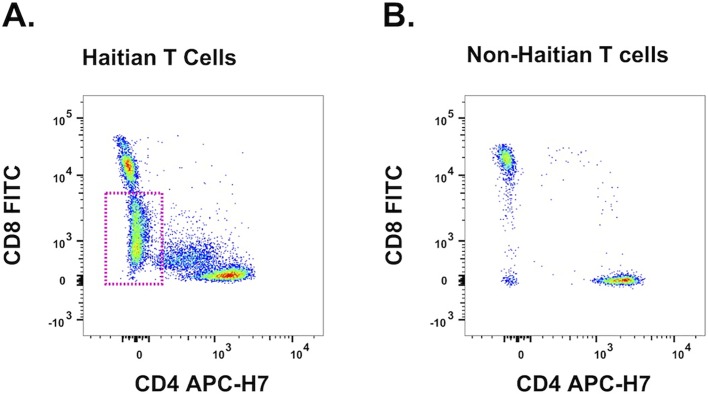
CD8^mid^ T cells are a unique haitian leukocyte population. PBMCs from all Haitian patients (n = 15) displayed a unique CD3^+^CD8^+^ T cell population, which expressed CD8, called CD8^mid^ T cells, at lower levels than non-Haitian individuals. **(A)** A representative flow cytometry dot plot of the CD4/CD8 axis of CD3+ T cells. The CD8^mid^ population of T cells is highlighted in pink. **(B)** Representative CD4/CD8 axis plot of PBMCs from individuals of non-Haitian origin (n = 4), stained during the same experiment, using the same reagents. The CD8^mid^ population is conspicuously absent.

**Fig 4:**
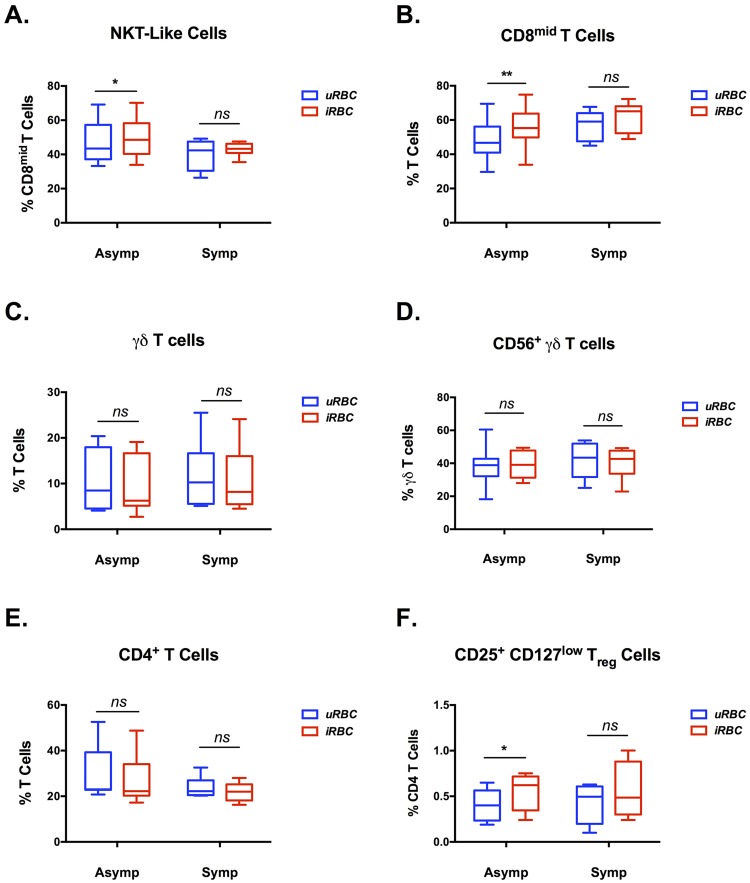
*P. falciparum* responsive T cell subtypes identify asymptomatic malaria patients. PBMCs from malaria patients were co-cultured for 6 days with lysate from uRBC or *P*. *falciparum* schizonts (iRBC lysate) at a lymphocyte to parasite ratio of 1:2. Changes in the percentages of T cell subpopulations during culture were compared between asymptomatic (n = 9, Asymp) and symptomatic (n = 6, Symp) patients in **(A)** NKT-like T cells, **(B)** CD8^mid^ T cells, **(C)** γδ T cells, **(D)** CD56^+^ γδ T cells, **(E)** CD4^+^ T cells, and **(F)** CD25^+^ CD127^low^ T regulatory cells. Data are represented as box and whisker plots, with box ends extending from the first to the third quartile and the median in the center. Whiskers extend to the highest and lowest data point. Statistical significance was determined using a two-tailed Wilcoxon matched-pairs signed rank test (iRBC lysate vs. uRBC for each patient). * = p-value < 0.05, ** = p-value < 0.01.

All Haitian patient PBMC samples, regardless of symptomatology, contained a population of T cells, herein referred to as CD8^mid^, expressing CD8 at variable levels which were conspicuously lower than non-Haitian individuals (Fig 3A and 3B). Triplicate Results from flow cytometric analysis unexpectedly revealed that 46.9% (mean, n = 15) of these CD8^mid^ cells expressed the prototypic NK cell maker, CD56 **(**S1 Fig, **cell population 9)**. Abnormal lymphocyte subtypes have been previously reported in patients from malaria endemic areas [10, 36]; however, at the time of writing, this phenotype has not been reported in Haiti to the best of our knowledge, and may be unique to the study population. Both CD8^mid^ populations (CD56^+^ and CD56^-^) experienced a statistically significant rise in the percentage of positive cells in the asymptomatic patient group exposed to infected erythrocytes at the schizont stage (iRBC lysate) vs uninfected erythrocytes (uRBCs; Fig 4A and 4B). This increase was not observed in the symptomatic group.

**Fig 5:**
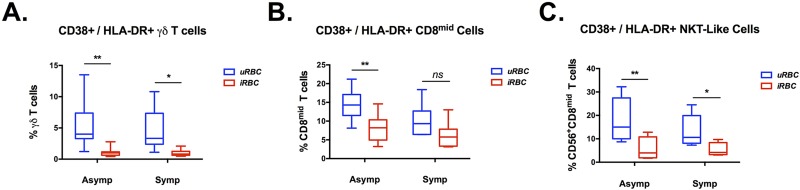
Analysis of CD38^+^/HLA-DR^+^ activation status of T cell sub-populations in asymptomatic and symptomatic malaria patients. Co-expression of CD38 and HLA-DR on T cells has been suggested as a marker of activation for malaria reactive T cells. The expression of these surface markers was analyzed in T cell subpopulations from asymptomatic (n = 9) and symptomatic (n = 6) patient PBMCs during exposure to *P*. *falciparum* strain H1064 infected erythrocytes (iRBC lysate) or uninfected erythrocytes (uRBC). Exposure to *P*. *falciparum* antigens diminished the activation status of γδ T cells **(A)**, CD^mid^ T cells **(B)**, and NKT-like T cells **(C)**. Statistical significance was determined using a two-tailed Wilcoxon matched-pairs signed rank test (iRBC lysate vs. uRBC for each patient). * = p-value < 0.05, ** = p-value < 0.01.

There was no increase in the percentage of γδ T cells in any of the patient groups when comparing co-culture with iRBC lysate to those with uRBC, nor was there an increase in γδ T cells expressing CD56, a subset of cells previously described as responsive to malarial antigens [10] (Fig 4C and 4D). Although there was no change in conventional CD4^+^ T cells populations in any group, we did observe a statistically significant rise in CD4^+^ CD25^+^ CD127^low^ T regulatory cells in the asymptomatic patients (Fig 4E and 4F). CD38^+^/HLA-DR^+^ expression on T cells has been proposed as a marker of activation in malaria immune responses [38]. We investigated expression of these markers on γδ T cells, CD8^mid^ cells, and CD8^mid^CD56^+^ NKT-like cells during our co-culture experiments. Results revealed that there was a statistically significant decrease in cell populations expressing these markers in the PBMCs exposed to iRBC lysate versus those exposed to uRBC in all three cell populations (Fig 5A–5C).

### Cytometric bead analysis of chemokines/cytokines produced by symptomatic and asymptomatic patient PBMCs co-cultured with *P*. *falciparum* schizont lysate

We hypothesized that in addition to the unique cellular responses observed using flow cytometry, the production of cytokines may further delineate immunological differences between the symptomatic and asymptomatic malaria patients in our cohort. In order to test this hypothesis we analyzed the supernatant from patient PBMC: Pf schizont lysate co-culture obtained from the flow cytometry experiments for the presence of chemokines/cytokines using cytometric bead technologies. We assayed the supernatants for the presence of 44 cytokines using the Luminex 200 platform.

**Fig 6:**
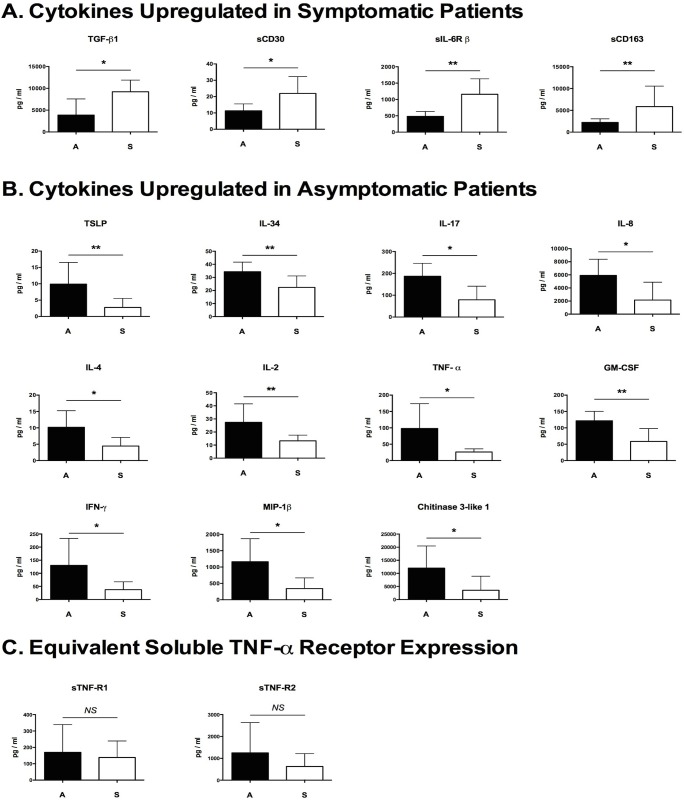
*P*. *falciparum*-infected erythrocyte lysate co-culture induces reciprocal cytokine expression profiles in symptomatic and asymptomatic malaria patient PBMCs. PBMCs from symptomatic (n = 6) and asymptomatic (n = 9) malaria patients were co-cultured with lysate from *P*. *falciparum* strain H1064 schizont infected erythrocytes at a schizont to effector cell ratio of 2:1 for 6 days and the resulting culture supernatant was assayed for cytokine concentration using multiplex analysis. This revealed a set of 15 cytokines that were reciprocally and differentially expressed in the patient groups. **(A)** one cytokine, a soluble cytokine receptor, and two soluble CD markers were more highly expressed by the symptomatic patient PBMCs. **(B)** 11 chemokines/cytokines were more highly expressed by asymptomatic patient PBMCs. **(C)** Asymptomatic patients produce more TNF-α when exposed to *P*. *falciparum* strain H1064 schizont lysate than symptomatic patients; however, both patient groups produce equivalent amounts of both soluble TNF-R1 and TNF-R2. Statistical significance was established using a two-tailed Mann Whitney test comparing the two patient groups. * = p-value < 0.05, ** = p-value < 0.01.

**Fig 7:**
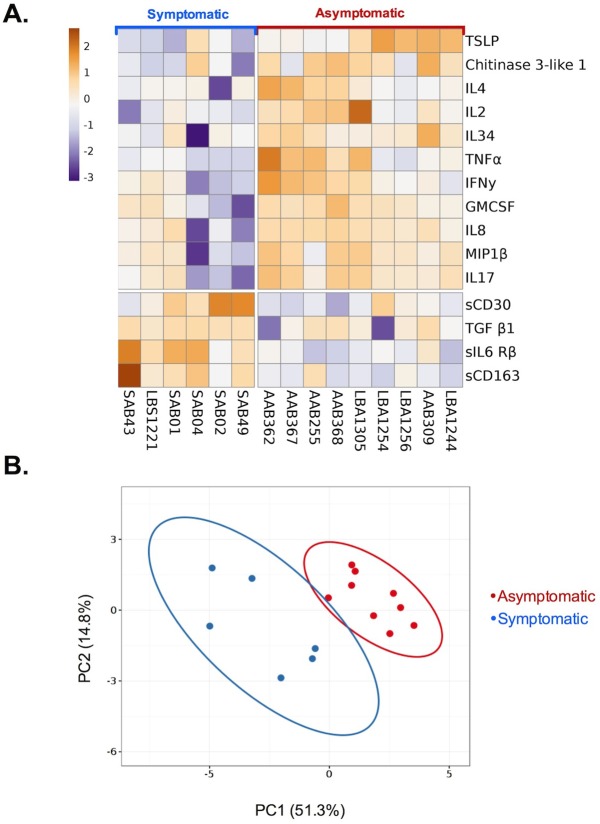
Heat map and principal component analysis of reciprocally expressed cytokines. **(A)** A heat map was generating using log_2_-transformed concentration values of 15 differentially expressed cytokines identified using multiplex analysis to visualize differences between symptomatic and asymptomatic malaria patient groups. Values were centered by subtracting the mean cytokine concentration from all samples from each individual data point. **(B)** Scatter plot of the first two principal components (comprising 66.1% of total variance) calculated using singular value decomposition (SVD) with imputation. Symptomatic patient values (blue closed circles) and asymptomatic patient values (red closed circles) cluster separately. Colored ellipses denote calculated 95% confidence levels of new observations from other patients from this population structure (symptomatic vs. asymptomatic) falling within the respective regions. There is minimal overlap between the confidence levels between patient populations. Both the heat map and PCA were constructed using ClustVis [39].

Results from this investigation demonstrated that there were 15 cytokines that were differentially expressed between patient groups at statistically significant levels. These included 4 cytokines that were expressed at higher levels in symptomatics: soluble CD30 (sCD30), TGF-β1, soluble IL-6 receptor β (s-IL6R β), and soluble CD163 (sCD163), (Fig 6A). Furthermore, 11 cytokines were upregulated in the asymptomatic patients: Thymic stromal lymphopoietin (TSLP), Chitinase-3-like 1 protein, IL-2, IL-4, IL-8, IL-17, IL-34, TNF-α, IFN-γ, GM-CSF, and MIP-1β (Fig 6B). Multiplex cytokines analysis also revealed that there likely exists aberrant TNF-α signaling capacity in the symptomatic patients. TNF-α secretion by PMBCs was significantly higher in the asymptomatic cohort, yet the expression of both soluble forms of its receptor, sTNF-R1 and sTNF-R2, were equivalent between the two patient groups (Fig 6C). Data from the 15 differentially expressed cytokines were then used to construct a heat map (Fig 7A), to visually demonstrate the reciprocal expression patterns of these signaling molecules between the two patient groups.

## Discussion

The goal of this comprehensive, cross-sectional investigation was to identify adaptive immunological differences between known asymptomatic and symptomatic *P*. *falciparum* patients from the Sud-Est department of Haiti. Our observations demonstrate that asymptomatic and symptomatic individuals were not differentiated by their humoral responses, but instead by their cellular and chemokine/cytokine responses. This is the first investigation where these cellular and chemokine/cytokine responses have been described in concert. Prior to this, only individual immune parameters have been described.

### IgG and IgM serological reactivity to *P*. *falciparum* antigens did not delineate between symptomatic and asymptomatic malaria patients from Haiti

The importance of humoral immunity during Pf infection has been appreciated since the discovery that passive antibody transfer from malaria immune individuals to naïve patients protects against the clinical forms of the disease [40]. Humans living in endemic areas gradually develop broad antibody repertoires to malarial antigens as they age, which is correlated with decreased severity of disease symptoms [16, 41]. This is particularly true when comparing antibody responses between uncomplicated malaria and severe malaria patients [42] as well as those comparing asymptomatic and symptomatic malaria patients.

This search for particular antibody-antigen complexes that discriminate asymptomatic vs. symptomatic malaria patients is an active area of research in the field [43], and one that has not yet been fully elucidated. In this investigation, we not only investigated the IgG activity against these antigens, but the IgM activity as well. IgM was included in this analysis, as it has been shown to provide protection during recurrent Pf infections through binding antigen targets and improving opsonization by dendritic cells and macrophages [44, 45]. The magnitude of both IgG and IgM responses to the selected Pf proteome in this cross-sectional study revealed no significant statistical differences between the two groups. Thus, no obvious target epitope was identified to define the phenotype of either population. These results were similar to those recently reported by Fratus *et al* [18], which found no diagnostic serological differences between asymptomatic and symptomatic Pf malaria patients in the Brazilian Amazon, which like Haiti, is an area of low disease transmission. Several proteins previously explored in studies that have been conducted in Africa [22, 46, 47] that demonstrate protection in those individuals to well documented Pf antigens as well as potential humoral vaccine targets were observed in the top twenty antigens based on magnitude in both of our patient cohorts (Tables 1 & 2). Merozoite surface proteins (MSP-1, MSP-2 and MSP-10) are attachment proteins expressed on the surface of the merozoites, critical for entry into host erythrocytes [22]. Early transcribed membrane proteins (ETRAMPs) are a family of *Plasmodium* transmembrane proteins that extrude into the erythrocyte cytoplasm from a vacuole created by the parasite during erythrocytic life stages [23, 48]. Interestingly, Liver Stage Antigens (LSA-1 and LSA-3) were also noted to have robust antibody responses; these proteins are expressed solely during hepatic stages, and are thought to be associated with merozoite development within hepatocytes [24, 49]. LSA-1 was the only protein simultaneously noted in both the top twenty IgG and IgM antigen responses. Since this is the first time that a protein microarray has been used to detect humoral immune responses in malaria-infected Haitian patients, it is difficult to make substantial conclusions. However, both the Haitian population and the local *P*. *falciparum* parasite are descended from western Africa, and have been coexisting for several hundred years with a low endemicity compared to Africa. This along with our data would suggest that this difference in phenotype is due to both a difference in endemicity of the parasite as well as a difference in the immune mechanisms (which this evidence suggests was not entirely humoral immunity).

Our observations demonstrated that there was evidence of cognate antibody recognition of the same Pf antigens in both groups, an absolute essential requirement of malaria immunity. However, the statistical equivalence of these responses was unable to account for the phenotypic differences between asymptomatic and symptomatic patients, indicating that the humoral response to Pf antigens is only a component of immune control in Pf infection. This was the impetus to further investigate cellular and chemokine/cytokine phenomena that could define both the asymptomatic and symptomatic phenotypes.

### *P*. *falciparum* responsive T cell subtypes unique to asymptomatic patients

During co-culture with Pf H1064 schizont infected erythrocyte lysate (iRBC lysate) we observed increased percentages of several T cell sub-populations in the asymptomatic patient cohort that did not occur with uninfected erythrocyte (uRBC) co-culture, nor in the symptomatic patients.

One subtype was a population of CD8^+^ T cells (Fig 4B). There is scant data in the scientific literature regarding the role these cells play during the blood stages of human malaria, reviewed in [50]. Despite the obvious lack of antigen presenting MHC I molecules on parasite infected erythrocytes, there are several reports detailing not only the generation of CD8^+^ T cells in response to blood stage *Plasmodium* infection [51, 52], but more importantly their contributions to protective immunity in murine models of malaria [53–55]. These cells were functional, pathogen-specific cytotoxic T lymphocytes producing granzyme B, perforin, TNF-α, and IFN-γ, which prime macrophages for enhanced phagocytosis of infected erythrocytes. Our results indicate that a similar phenomenon is occurring within the asymptomatic human subjects in our cohort, and the lack of a similar response in the symptomatic patients suggests that CD8^+^ T cells may be involved in a protective immune response.

A second subtype of responsive cells was a population of CD3^+^CD8^+^CD56^+^ NKT-like cells (Fig 4A). The function of these cells in context of malaria remains unexplored, but studies in other disease models have established these cells as cytolytic and capable of secreting Th1 inflammatory cytokines [56]. For example, these NKT cells are critical effectors in the maintenance of long-term non-progressor status in HIV-infected individuals [57]. Interestingly, NKT-like cells have also recently been reported to suppress over-activated immune processes through killing of antigen bearing dendritic cells [58]. Certainly, further investigations are required to explore the role of these cells in the human immune response to malaria. Interestingly, we observed that IL-2 and TSLP were more highly expressed in asymptomatic patients from our cohort (Fig 5B). Since the differentiation and persistence of effector phenotypes in both NKT-like and CD8^+^ T cells, are positively regulated by these mediators [59, 60], they may be of particular interest for further inquiry.

An important aspect of clinical immunity to malaria is the ability to dampen the inflammatory response after control of parasitemia is established in order to avoid immune-mediated pathology. T regulatory cells are known as key mediators of immune homeostasis and both increase during human malaria as well as modulate the host response to infection [61]. Our results showed a CD4^+^CD25^+^CD127^low^ T regulatory cell sub-population that increased in frequency upon exposure to Pf schizonts in asymptomatic patients was (Fig 4F); this was an interesting point, since there have been recent investigations into the role that T regulatory cells play in the symptomology of malaria, which contrast with our results. These current studies from African cohorts have documented both a decrease in overall T regulatory cells numbers in Ugandan children with recurrent exposure to Pf malaria [27], as well as an association between a variant FOXO3A polymorphism in Gabonese children with severe Pf malaria [62]. Furthermore, a similar phenomenon was described in a longitudinal study following asymptomatic malaria patients in The Gambia. They reported that rises in Th1 immune responses during the transmission season were concurrently matched with increases in T regulatory cells [63], implying a tight co-regulation of effector and regulatory cells during the course of infection.

### Down regulation of T cell activation markers in both symptomatic and asymptomatic patient PBMCs

We observed a decrease in the expression of both CD38 and HLA-DR in γδ, NKT-like, and CD8^+^ T cells in PBMCs from both patient groups exposed to Pf antigens in iRBC lysate, compared to those incubated with uRBC, (Fig 5A–5C). All of these decreases were statistically significant except for the CD8^+^ T cells from symptomatic patients. The cause of this down regulation could not be ascertained, and it remains to be determined if this cellular phenotype is the result of an unknown immuno-modulatory process of the host or from a parasite-derived factor. It is worth noting that this same phenomenon of decreased HLA-DR expression was recently observed in monocytes obtained from Malawian children presenting at hospital with malaria [64], suggesting that this process may affect all leukocytes. Further research is certainly needed before any further inferences could be drawn.

### Monocyte activating cytokines are upregulated in asymptomatic patients

We identified 11 cytokines and chemokines that were expressed at higher concentrations in asymptomatic compared to symptomatic malaria patients within our cohort (Fig 6B). Some of these molecules have previously been described in the literature as promoting the activation and persistence of cells of the monocyte lineage, or are themselves markers of differentiation. Granulocyte macrophage colony stimulating factor (GM-CSF) is a potent activator of differentiation, cytokine expression, phagocytosis, surface receptor expression, and oxidative metabolism in monocytes [65] which has been implicated in protection from malaria in both human patients and murine models [12, 14, 66]. Similarly CCL4, also known as macrophage inflammatory protein 1β (MIP-1β), synthesized by CD8^+^ T cells, is a strong chemoattractant for monocytes. Its expression too has been linked to malaria infection and *Plasmodium* metabolites [67, 68]. Our results also indicated that IL-34 was upregulated in asymptomatic patients. Although there is an increasing appreciation for the tolerogenic effects of this cytokine on T cell populations [69], its original discovery elucidated its stimulatory effect on monocyte viability via signaling through the M-CSF receptor [70]. In addition, the upregulated IL-17 and IFN-γ both activate macrophages and prime them for phagocytosis [71, 72]. Although there is experimental evidence that IL-34 expressed by T regulatory cells can induce a suppressive phenotype in leukocytes [69], the cytokine has also been implicated in pro-inflammatory processes associated with rheumatoid arthritis ([73]), where IL-34 expression has been recently shown to be inhibited by TGF-β1 [74], which induces an immunosuppressive effect within the synovium. Its effects are therefore context dependent and it should be noted that high levels of IFN-γ and GM-CSF as seen in the asymptomatic population (Fig 6B) are known to antagonize the immunosuppressive effects of IL-34 in macrophages [75]. Finally, the secretion of TNF-α, IL-8, and thymic stromal lymphopoietin (TSLP) are all known markers of an activated phenotype in macrophages [76–78].

The identification of monocyte/macrophage activating cytokines may have implications in the understanding of a particular feature of malarial immunology known as premunition or infection-immunity [79]. After numerous Pf exposures, equilibrium is reached with a chronic stage of infection of the host. Premunition is seen in endemic areas of disease and is characterized by a non-sterilizing immunity with lower parasite loads and a substantial reduction in morbidity. Early experiments established that passive transfer of immune sera to naïve individuals drastically reduced symptoms of acute infection indicating that antibodies played a central role in premunition [40, 80, 81]. Later it was shown that this immunological phenomenon required cooperation between antibodies and monocytes in order to be effective [41, 82, 83]. Patient data from Malawi has recently demonstrated that in patients with severe malaria, monocytes have decreased expression of activation markers compared to those from patients with uncomplicated malaria, underscoring the role that activated monocytes play in controlling morbidity [64].

In this study we observed a statistically equivalent repertoire of antibodies in our asymptomatic and symptomatic patients, both in terms of the breadth and magnitude of the response. This indicates that serological differences likely do not drive immune differences between our cohorts. Since premunition requires monocyte cooperation as well as antibody to Pf antigens, perhaps the elevated levels of cytokines and chemokines known to activate cells of the monocytic lineage observed in our asymptomatic patients serve to more efficiently prime these cells for phagocytosis and clearance of the parasite. Given that premunition is the strongest type of immunity mounted by humans against the asexual erythrocytic life stages of the parasite [79], these new data may at least partially explain the differences in clinical morbidity observed within our cohort.

### Immuno-modulatory soluble mediators are upregulated in symptomatic patients

Our multiplex cytokine analysis showed that four soluble factors were produced at higher concentrations in the culture supernatants of symptomatic patient PBMCs vs. asymptomatic PBMCs during co-culture with Pf asexual blood stage antigens (Fig 6A).

Expression of sCD30 was significantly elevated in our symptomatic patient cohort compared to the asymptomatic cohort (Fig 6A). The soluble form of CD30 (sCD30) is a cleavage product of the extracellular domain of the full-length membrane bound CD30, which is expressed by activated lymphocytes and cleaved by the action of metallo-proteinases [84]. Membrane bound CD30-CD30L signaling promotes the activation of NF-κB and the production of Th2 type cytokines from CD4^+^ and CD8^+^ T cells [85]; however, increased production of sCD30 leads to an over-activation of this pathway and has been linked to several Th2 mediated diseases including systemic lupus erythematosus, rheumatoid arthritis, atopic dermatitis, and Omenn’s syndrome [84–86]. Elevated levels of sCD30 in malaria patients has been reported elsewhere [13], and although further investigations are necessary, it is tempting to speculate that this protein may exacerbate inflammatory processes leading to increased morbidity. This may be of particular import as abrogated Th2 immune responses have been positively correlated with asymptomatic malaria infections [87].

Data from our current study indicating an elevated expression of sCD163 by PBMCs from symptomatic malaria patients may explain some of their susceptibility to the disease, as it has been demonstrated that plasma levels of sCD163 are inversely correlated with CD163 expression on monocytes [88]. Intravascular hemolysis is a hallmark of the immunopathology of Pf malaria that results in increased concentrations of free hemoglobin. The soluble form of the CD163 receptor (sCD163) antagonizes the clearance of heme from circulation and is associated with inflammatory processes. Our results are in agreement with those of Mendonça *et al* who showed that increased plasma concentrations of sCD163 were associated with increased susceptibility to malaria [89].

The expression of sIL-6Rβ was much higher in our symptomatic patient cohort than in the asymptomatic one (Fig 6A). While there is a paucity of data on this receptor in the literature with regards to human malaria, blockade of trans-signaling by IL-6, of which sIL-6Rβ is the crucial mediator, has been shown to protect against malaria-induced lethality in mice [90]. The soluble form of the IL-6 receptor (sIL-6Rβ) has been shown to promote the retention of activated mononuclear cell populations within inflamed tissues [91] via trans-signaling with IL-6, which can lead to maintenance of the disease state and a transition to chronic inflammation [92, 93]. Our study is the first to report a connection between sIL-6Rβ expression and disease status in human malaria.

Our observation that PBMCs from the symptomatic cohort have higher levels of TGF-β1 than patients from the asymptomatic cohort (Fig 6A) suggests that early induction of high levels of TGF-β1 expression are anti-inflammatory and create an environment permissive to the replication of the *Plasmodium* parasite. Transforming growth factor β1 is an important regulator of inflammation, as well as a key mediator between clearance of infectious organisms and immune-mediated pathology [94]. Its effects vary depending on the concentration and temporal expression. At low concentrations TGF-ß is pro-inflammatory while at high concentrations it is anti-inflammatory. In a murine model of malaria, early administration of high amounts of TGF-β1 resulted in the failure of resistance in a mouse strain normally immune to infection [95]. In human malaria, similar results have been reported with high TGF-β1 expression being linked to increased parasite growth *in vivo* [96] and disease severity [97, 98]. In addition TGF-β1 is known to suppress the expression of both TNF-α and IFN-γ ([94], which are key cytokines necessary for early control of the acute phase of *Plasmodium* infection [72, 97, 99].

PBMCs from our symptomatic cohort expressed significantly higher levels of soluble receptors and cytokines, each of which has individually been associated with poor disease outcomes, compared to PBMCs from asymptomatic patients. This is the first report in the literature to describe the combinatorial contributions of all 4 molecules (sCD30, sCD163, sIL-6Rβ, TGF-β1) to promoting susceptibility to Pf malaria.

### Study limitations

This investigation was not without limitations in the following areas. We recognize that a total of twenty-nine patients who were enrolled in the study demonstrates a lower statistical power; this can be explained by the following. Sud-est is a highly mountainous area of Haiti with the exception of the Caribbean Sea coast, which offers a smaller area for populations with endemic malaria. Also, local community leaders in Haiti requested that a limited amount of whole blood be phlebotomized from patients (<50mL), and despite this due to local customs, most individuals who were in the documented in previous malaria cohort studies, refrained from participating due to the amount of required phlebotomized blood. This not only reduced the amount of patients that participated, but subsequently limited the quantity of plasma and PBMCs collected as well. Therefore, repeating cell culture and subsequent flow cytometry and multiplex analysis was not an option, which only provided a single triplicate data set for these experiments. Despite the fact that the country of Haiti is relatively small in area, the topography made travel between village sites and the processing of laboratory samples cumbersome multi-day events. The fact that this type of analysis has not previously been performed is in no small part due to the sheer difficulty of obtaining and securing plasma and PBMCs from such remote areas. Also, because of the limited number of patient cells, there were a limited number of extracellular and intracellular (i.e. Foxp3^+^) markers (or even Foxp3^+^ polymorphisms noted in Africa) that could have been investigated using flow cytometry or deep sequencing, which is why we designed experiments that examined T cell subpopulations and chemokines/cytokines simultaneously.

All cellular population changes that are reported in this study were thus linked to concurrent inflammatory mediator expression profiles. It should be noted however, that the cell types responsible for the secretion of these cytokine repertoires were not determined and that future experiments are needed to further resolve these observations. For instance, asymptomatic patients had increases in Treg cells as well as a statistical increase in IL-34, as well as a statistical increase in TGF-β1. However, the signature cytokines of these cells, IL-10 (IL-10 levels were at the lower limit of detection for both cohorts) and IL-35 (S2 Fig), and furthermore, IL-10 and IL-35 were expressed at statistically equivalent levels between asymptomatics and symptomatics.

Pf protein microarray antigens are created using a cell-free *E*. *coli* lysate system, and thus do not contain any antigenic glycans normally added by Pf to exported proteins during post-translational glycosylation. Therefore, the breadth of the antibody target repertoires that were elucidated by the protein microarrays is not completely definitive due to the lack of these antigenic alterations. Additionally, due to the high throughput nature of this technology, protein folding and conformation of B cell epitopes cannot be validated, although conformation and epitope-specific reagents for most proteins on the arrays remain unavailable for validation. Indeed, the discovery aspects of the technology may lead to identification of novel B cell antigens, as seen in previous studies [16, 20, 21, 26]. Thus, these results should be interpreted with some discretion.

## Conclusions

The results of this investigation demonstrate that asymptomatic and symptomatic individuals in our cohort were delineated by both their cellular and chemokine/cytokine responses, but not by their antibody responses. Many of these differentiating cellular and chemokine/cytokine responses have been singularly identified in many other studies, yet these results have not been reported in the comprehensive manner as was done in this investigation. Our results demonstrate two main points. 1) We described malaria responsive T cell subsets unique to asymptomatic patients, which corroborates evidence from other studies involving T regulatory cells [61], and we also provide preliminary evidence that human CD8+ T cells are reactive to asexual blood stage antigens, a phenomenon only previously reported in rodent malaria [50]. 2) In addition, we observed increased levels of monocyte-activating chemokines/cytokines in these asymptomatic malaria patients. The reciprocal expression pattern of these inflammatory mediators (Fig 6A and 6B) and principal component analysis (Fig 7B) suggest that they may be measurable and predictive correlates of disease state.

This study, along with others [100] from other malaria endemic areas of the world, using large scale biological data sets analyzing multiple components of adaptive immunity, will collectively assist in defining which cellular responses and molecular correlates of disease outcome are malaria region specific and furthermore, which are truly generalizable features of asymptomatic *Plasmodium* immunity, a research goal of critical priority [101].

## Materials and methods

### Study sites, study participants and ethical statement

Two study sites from the Sud-Est Department, La Brésilienne (18° 11' 32" N, 72° 39' 09" W) and Anse à Boeuf (18° 13' 00" N, 72° 00' 00" W) were used in this current study (Fig 1). Although there is a low prevalence of transmission, historically this department has consistently reported cases of malaria in Haiti. Previous studies in this geographical region had identified a cohort of *P*. *falciparum* (Pf) malaria patients, including several non-febrile asymptomatic infections [19, 102, 103]. Samples were collected between April 2015-June 2015. There were a total of 29 participants, with both men and women enrolled in the study from an age range of 18 to 64 years, with a mean age of 32.5 years. Participants were previously classified as having symptomatic or asymptomatic malaria infections. This was performed based on clinician diagnosis of parasitemia (using malaria rapid diagnostic test (RDT) designed to detect histidine-rich protein 2 (HRP2) and thick smear microscopy) in individuals who had signs of fever; individuals who were discovered to have asymptomatic malaria, were noted to have lived either in the same household or in the vicinity of the symptomatic patient, were not febrile and were positive for parasitemia [19, 102]. Samples were approximately evenly split between two villages in Haiti (Anse à Boeuf: n = 14; La Brésilienne: n = 15). Characteristics of the participants’ demographics are listed in Table 1. Malaria naïve individuals (n = 4) were recruited from healthy volunteers at the University of Florida.

This study was conducted in accordance with institutional review board guidelines and requirements of the University of Florida and the ethical review board of the Haitian MSPP under approved study protocols 201401022 and 1415–27; all participating subjects were provided written informed consent in accordance with both the University of Florida institutional review board as well as the ethical review board of the Haitian MSPP.

### Blood sample collection and preparation

At each of the study sites, venous blood was collected from consented participants into BD Vacutainer CPT Mononuclear Cell Preparation Tubes (BD Biosciences) with sodium citrate. Tubes with separated blood samples were centrifuged for 10 minutes at 2000 rpm on site, and then stored on ice for ~ 8 hours during transportation to the University of Florida research lab in Gressier, Haiti. Both plasma and PBMCs were then separated via further centrifugation for 10 minutes at 2000 rpm. Plasma was decanted and stored at -80°C. Cells were enumerated using 0.4% trypan blue (Thermo Fisher Scientific) and a hemacytometer (Hausser Scientific) and stored at -80°C in RPMI 1640 with L-glutamine and 25mM HEPES (Corning Mediatech) supplemented with 10% heat-inactivated Hi-FBS (Gibco Life Technologies) and Penicillin/Streptomycin solution (Gibco Life Technologies) with 10% molecular grade DMSO. Samples were shipped on dry ice via air courier to the Emerging Pathogens Institute at the University of Florida, Gainesville, FL. Upon arrival to the Emerging Pathogens Institute, plasma was stored at -80°C and PBMCs were stored in liquid nitrogen until used in experiments.

### Proteome microarray construction

Pf protein microarray technology was chosen for this analysis due to the vast number of Pf proteins that could be screened at one time compared to other methods. The partial proteome microarray developed at Antigen Discovery Inc., used in this study covered approximately 50% coverage of the Pf proteome, containing 3,877 Pf full-length proteins or peptide fragments (for genes > 3kb) representing proteins from 2,704 unique Pf genes that were selected iterative development of Pf proteome microarrays using protein annotation from PlasmoDB (www.plasmodb.org) for hypothesized immunoreactive erythrocytic stage (asexualsexual stages) and pre-erythrocytic stage proteins. Most known vaccine candidate and studied proteins were included on the array. The half-proteome array used in this study was selected for enrichment of erythrocytic stage proteins known to be targets of naturally acquired immunity [26] as well as unpublished data). Seropositivity was defined as at least 10% of the antibodies from the study populations recognizing target antigen with at least twice the signal intensity of the empty vector negative controls (a.k.a. background). Proteins were expressed using an *in vitro* transcription and translation (IVTT) system, the *Escherichia coli* cell-free Rapid Translation System (RTS) kit (5 Prime, Gaithersburg, MD, USA). A library of Pf partial or complete open reading frames (ORFs) cloned into a T7 expression vector pXI has been established at Antigen Discovery, Inc. (ADi, Irvine, CA, USA). This library was created through an *in vivo* recombination cloning process with PCR-amplified Pf ORFs, and a complementary linearized expressed vector transformed into chemically competent *E*. *coli* was amplified by PCR and cloned into pXI vector using a high-throughput PCR recombination cloning method described elsewhere [15]. Each expressed protein includes a 5’ polyhistidine (HIS) epitope and 3’ hemagglutinin (HA) epitope. After expressing the proteins according to manufacturer instructions, translated proteins were printed onto nitrocellulose-coated glass AVID slides (Grace Bio-Labs, Inc., Bend, OR, USA) using an Omni Grid Accent robotic microarray printer (Digilabs, Inc., Marlborough, MA, USA). Microarray chip printing and protein expression were quality checked for expression by probing random slides with anti-HIS and anti-HA monoclonal antibodies with fluorescent labeling.

### Proteome microarray chip design

The Pf proteome microarrays used in this study was a partial proteome microarray with approximately 50% coverage of the protein-coding Pf proteome. Each chip contained 3,877 Pf peptide fragments representing proteins from 2,704 unique Pf genes, 192 IgG positive control spots and 160 spotted IVTT reactions without Pf ORFs (IVTT controls). For each chip, 3 replicates were printed per microarray slide on 3 nitrocellulose “pads”. IgG positive control spots were included as an assay control, while IVTT control spots were included as a sample-level normalization factor.

### Proteome microarray sample probing

Serum samples were diluted 1:100 in a 3mg/mL *E*. *coli* lysate solution in protein arraying buffer (Maine Manufacturing, Sanford, ME, USA) and incubated at room temperature for 30 min. Chips were rehydrated in blocking buffer for 30 min. Blocking buffer was removed, and chips were probed with pre-incubated serum samples using sealed, fitted slide chambers to ensure no cross-contamination of sample between pads. Chips were incubated overnight at 4°C with agitation. Chips were washed five times with TBS-0.05% Tween 20, followed by incubation with biotin-conjugated goat anti-human IgG (Jackson ImmunoResearch, West Grove, PA, USA) diluted 1:200 in blocking buffer at room temperature. Chips were washed three times with TBS-0.05% Tween 20, followed by incubation with streptavidin-conjugated SureLight P-3 (Columbia Biosciences, Frederick, MD, USA) at room temperature protected from light. Chips were washed three times with TBS-0.05% Tween 20, three times with TBS, and once with water. Chips were air dried by centrifugation at 1,000 x g for 4 min and scanned on a ScanArray Express HT spectral scanner (Perkin-Elmer, Waltham, MA, USA), and spot and background intensities were measured using an annotated grid file (.GAL). Data were exported in Microsoft Excel.

### Proteome microarray data processing

Raw spot and local background fluorescence intensities, spot annotations and sample phenotypes were imported and merged in the R statistical environment (www.r-project.org), where all subsequent procedures were performed. Foreground spot intensities were adjusted by local background by subtraction, and negative values were converted to 1. Next, all foreground values were transformed using the base 2 logarithm (Log2). The dataset was normalized to remove systematic effects by subtracting the median signal intensity of the IVTT controls for each sample. Since the IVTT control spots carry the chip, sample and batch-level systematic effects, but also antibody background activity to the IVTT system, this procedure normalizes the data and provides a relative measure of the specific antibody binding to the non-specific antibody binding to the IVTT controls (a.k.a. background). With the normalized data, a value of 0.0 means that the intensity is no different than the background, and a value of 1.0 indicates a doubling with respect to background. A seropositivity threshold was established as twice the IVTT background, or a normalized signal of 1.0. Reactive antigens were defined as those that had seropositive responses in at least 10% of the study population, or 3 subjects. Non-reactive antigens were filtered before group comparisons were performed to reduce unneccessary statistical tests.

Quality control plots were made after each treatment of the data, which included boxplots and density plots of probe intensity by study sample and probe type. Two outcomes were tested in each group comparison: 1) antibody levels for each antigen, and 2) the count of seropositive responses for each individual (“antibody breadth”). In all statistical tests, P-values were adjusted for the false discovery rate using the Benjamini-Hochberg method [104] and a finding was considered significant for Benjamini-Hochberg-adjusted (BH) P-values less than 0.05. For antibody levels, antibodies for each antigen were tested by empirical Bayes moderated T tests [105] using the “limma” package in R, and results were compared with Student’s T-tests. Group-wise comparisons were displayed graphically using box plots. Effect estimates of antibody breadth score on the probability of having symptomatic malaria were performed using logistic regression.

### *P*. *falciparum* strain H1064 provenance and *in-vitro* culture

*P*. *falciparum* H1064 was collected on February 2, 2015 in Baradielle (Dame Marie), Ouest District of Haiti by blood draw from an infected patient (diagnosis confirmed using RDT) and returned to our lab for culture following the protocol of Nielsen and Staaloe [106]. The antigen prep was prepared as follows to obtain high concentration of schizont infected O^+^ erythrocytes free of most cellular debris. A fresh, semi-synchronous culture was prepared by the sorbitol method [107] with gentamicin (50μg/L); mature schizonts collected on a magnetic column [108]. The mature schizonts were released from the column and % iRBC and the total number of cells determined by microscopy and Nexcelom Cellometer count. Equivalent numbers of uninfected RBCs were also prepared by Cellometer count.

### Patient PBMC: *P*. *falciparum* infected erythrocyte lysate co-culture

Cryo-preserved PBMCs from 6 symptomatic, 9 asymptomatic, and 4 malaria naive patients were thawed and washed with ice cold PBS and then enumerated and assessed for viability using 0.4% trypan blue (Thermo Fisher Scientific) and a hemacytometer (viability ranged from 80–85%). 500,000 viable PBMCs from each patient were resuspended in 500 μl RPMI 1640 with L-glutamine and 25mM HEPES (Corning Mediatech) supplemented with 10% heat-inactivated Hi-FBS (Gibco Life Technologies) and Penicillin/Streptomycin solution (Gibco Life Technologies), and plated in a 24 well polystyrene tissue culture plate. Lysate (3x freeze/thaw cycles -80°C / 37°C) from 10^6^ *P*. *falciparum* strain H1064 shizont-infected erythrocytes (iRBC lysate) or an equivalent quantity of uninfected erythrocytes (uRBC) were added to the wells at an erythrocyte: PBMC ration of 2:1 to reflect a parasitemia range of natural infection (2:1 ~0.1% assuming 6 x 10^6^ erythrocytes and 3 x 10^3^ PMBCs per μl of whole blood).

### Cell phenotyping by flow cytometry

Cell surface staining of study PBMCs was performed using fluorophore-conjugated antibodies against the following proteins: CD45-V450 (HI30, BD Biosciences), CD3-V500 (SP34-32, BD Biosciences), TCR γδ-PE (B1, BD Biosciences), CD8-FITC (HIT8a, BD Biosciences), CD4-APC H7 (SK3, BD Biosciences), CD56-PE Cy7 (B159, BD Biosciences), HLA-DR-APC (TU36, BD Biosciences), CD127-APC R700 (HIL-7R-M21, BD Biosciences), CD25-PE CF594 (M-A521, BD Biosciences) CD38-Qdot655 (HIT2, Thermo Fisher Scientific). Briefly, Cells were washed with PBS, incubated with human TruStain FcX (Biolegend) to block Fc receptors, and then incubated with antibodies diluted in stain buffer (BD Pharmigen) for 2 hours at 25°C. After staining cells were washed twice with PBS and fixed with 2% paraformaldehyde. Following staining, events were collected using a LSR Fortessa flow cytometer (BD Biosciences) and fluorophore compensation was calculated using single stained PBMC controls on the BD FACSDiva software package. Resulting Data were analyzed using FlowJo Software (Tree Star) and GraphPad Prism (GraphPad Software). Statistically significant differences in PBMC cell population percentages between iRBC lysate and uRBC co-cultures for each patient group (symptomatic vs. asymptomatic) were identified using a two-tailed Wilcoxon matched pairs signed rank test. P-values of < 0.05 were considered statistically significant.

### Multiplex cytokine expression analysis

Cell culture supernatants collected from PBMC (n = 9 asymtomatics, n = 6 symptomatics) co-culture with lysates of *P*. *falciparum* infected erythrocytes and stored at -80°C until use, samples underwent one freeze/thaw cycle. Supernatants were analyzed for cytokine production on a Luminex 200 analyzer (Luminex) using the following assays [all from Bio-Rad]: Bio-plex Pro TGF-β assay [#171X40501] (TGF-β1, TGF-β2, TGF-β3) [, Bio-plex Pro human inflammation panel I assay (APRIL, BAFF, sCD30, sCD163, Chitinase-3-like 1, gp130, IFN-β, IL-11, IL-19, IL-20, IL-26, IL-27, IL-28A, IL-29, IL-32, IL-35, LIGHT, Osteocalcin, Pentraxin-3, sTNF-R1, sTNF-R2, TSLP, TWEAK) [#171AL002M], and Bio-plex cytokine, chemokine, growth factor assay (G-CSF, GM-CSF, IFN-γ, IL-1β, IL-2, IL-4, IL-5, IL-6, IL-7, IL-8, IL-10, IL-12, IL-13, IL-17, MCP-1, MIP-1β, TNF-α) [#M5000031YV]. Assays were performed according to manufacturer’s instructions. Briefly, culture supernatants and cytokine capture-bead cocktails were incubated for 2 hrs, incubated with biotin-labeled anti-cytokine for 1.5 hrs and then with in a streptavidin-phycoerythrin reporter for 30 minutes. Data were analyzed using Milliplex Analyst software (Millipore) with 5 parameter logistics and standard curves, Excel (Microsoft), and GraphPad Prism (Prism Software). Statistically different concentrations of cytokines were identified using a two-tailed Mann Whitney test, p-values of < 0.05 were considered statistically significant.

### Principal component and heat map analysis

Cytokines that were identified as being differentially expressed at statistically significant levels in PBMCs from symptomatic and asymptomatic patients were log_2_ transformed into an array using Excel (Microsoft). These data were comprised of experimental measurements of 15 cytokine concentrations: TGF-β1, sCD30, sCD163, sIL-6Rβ, TSLP, IL-34, IL-17, IL-8, IL-4, IL-2, GM-CSF, TNFα, Chitinase 3-like 1, IFNγ, and MIP-1β. Principal components were calculated using singular value decomposition (SVD) with imputation followed by unit variance scaling using ClustVis [39] (http://biit.cs.ut.ee/clustvis/, accessed 11 May 2016). A scatter plot with the axes corresponding to the first two principal components was generated with prediction ellipses at a 95% confidence level delineating symptomatic and asymptomatic patient groups. ClustVis was also used to generate a heat map of cytokine levels from each of the 15 patient samples. Values were centered by subtracting the mean chemokine/cytokine concentration from all samples from each individual data point.

## Supporting information

S1 Fig Gating strategy for flow cytometry analysis. The schematic is a representative PBMC analysis whereby gated populations are indicated as defining: **(1)** Live leukocyte population based on FSC and SSC characteristics. **(2)** Total T cell population based on CD45^+^ CD3^+^ expression. **(3)** Non-Gamma Delta T cells and **(4)** Gamma Delta T cells based on expression of the γδ TCR. **(5)** Non-CD4^+^ and **(6)** CD4^+^ cells based on expression of CD4. **(7)** Cells expressing a high level of CD8 and **(8)** Cells expressing a mid-level of CD8. **(9)** CD56^+^ CD8^mid^ cells or **(10)** CD56^-^ CD8^mid^ cells. **(11)** CD38^+^/HLA-DR^+^ populations were identified from both CD56^+^ and CD56^-^ CD8^mid^ populations. **(12)** CD25^+^/CD127^low^ T regulatory cells from the population of CD4^+^ cells. **(13)** CD56^+^ Gamma Delta T cells. **(14)** CD38^+^/HLA-DR^+^ population of Gamma Delta T cells. Positive gates were established using fluorescence minus one (FMO) controls.
(TIF)

S2 Fig IL-35 expression profiles of P. falciparum-infected erythrocyte lysate co-culture in symptomatic and asymptomatic malaria patient PBMCs. As noted in Fig 6, PBMCs from symptomatic (n = 6) and asymptomatic (n = 9) malaria patients were co-cultured with lysate from P. falciparum strain H1064 schizont infected erythrocytes at a schizont to effector cell ratio of 2:1 for 6 days and the resulting culture supernatant was assayed for cytokine concentration using multiplex analysis. IL-35 was expressed at statistically equivalent levels between asymptomatics and symptomatics. Statistical significance was established using a two-tailed Mann Whitney test comparing the two patient groups. * = p-value < 0.05, ** = p-value < 0.01.
(TIF)
