# Supplementary figures and images for: T cell subtypes and reciprocal inflammatory mediator expression differentiate P. falciparum memory recall responses in asymptomatic and symptomatic malaria patients in southeastern Haiti
Source: PLoS One. 2017 Apr 3;12(4):e0174718. doi: 10.1371/journal.pone.0174718 (PMC5378365; doi:10.1371/journal.pone.0174718)

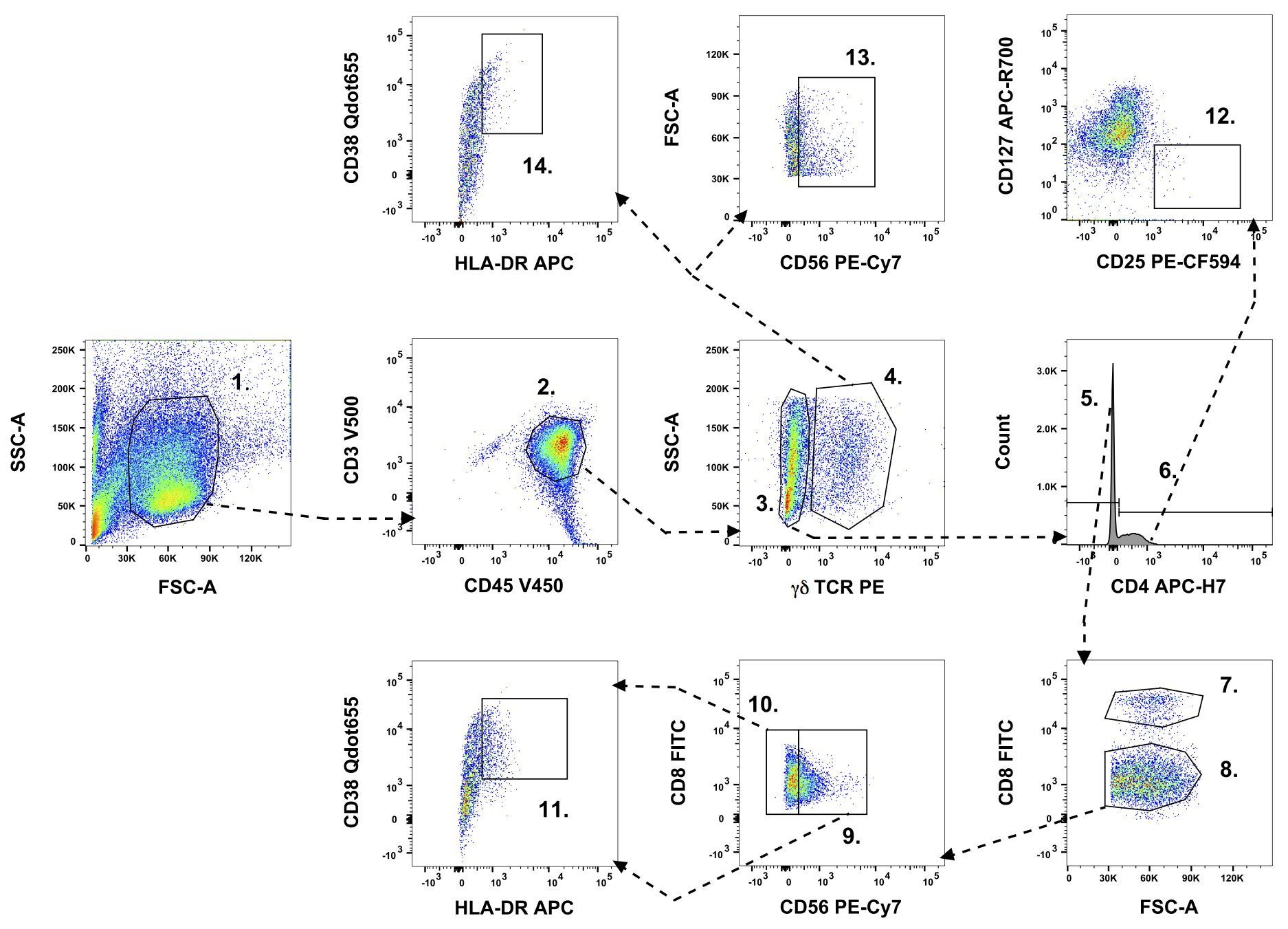

Supplement: S1 Fig — The schematic is a representative PBMC analysis whereby gated populations are indicated as defining: (1) Live leukocyte population based on FSC and SSC characteristics. (2) Total T cell population based on CD45+ CD3+ expression. (3) Non-Gamma Delta T cells and (4) Gamma Delta T cells based on expression of the γδ TCR. (5) Non-CD4+ and (6) CD4+ cells based on expression of CD4. (7) Cells expressing a high level of CD8 and (8) Cells expressing a mid-level of CD8. (9) CD56+ CD8mid cells or (10) CD56- CD8mid cells. (11) CD38+/HLA-DR+ populations were identified from both CD56+ and CD56- CD8mid populations. (12) CD25+/CD127low T regulatory cells from the population of CD4+ cells. (13) CD56+ Gamma Delta T cells. (14) CD38+/HLA-DR+ population of Gamma Delta T cells. Positive gates were established using fluorescence minus one (FMO) controls. (TIF) [file pone.0174718.s001.tif]

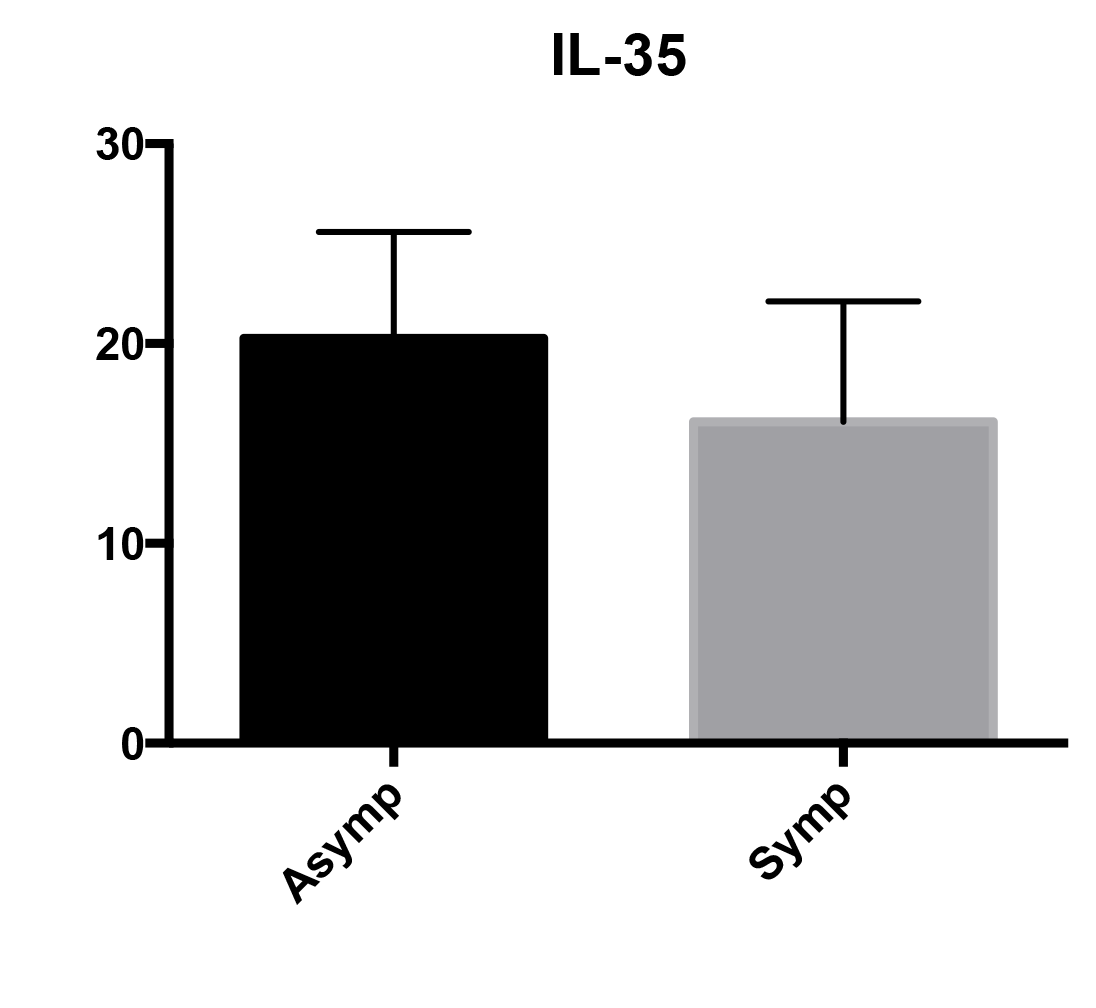

Supplement: S2 Fig — As noted in Fig 6, PBMCs from symptomatic (n = 6) and asymptomatic (n = 9) malaria patients were co-cultured with lysate from P. falciparum strain H1064 schizont infected erythrocytes at a schizont to effector cell ratio of 2:1 for 6 days and the resulting culture supernatant was assayed for cytokine concentration using multiplex analysis. IL-35 was expressed at statistically equivalent levels between asymptomatics and symptomatics. Statistical significance was established using a two-tailed Mann Whitney test comparing the two patient groups. * = p-value < 0.05, ** = p-value < 0.01. (TIF) [file pone.0174718.s002.tif]
